# Discovery of novel candidates for anti-liposarcoma therapies by medium-scale high-throughput drug screening

**DOI:** 10.1371/journal.pone.0248140

**Published:** 2021-03-10

**Authors:** Iwona Grad, Robert Hanes, Pilar Ayuda-Durán, Marieke Lydia Kuijjer, Jorrit M. Enserink, Leonardo A. Meza-Zepeda, Ola Myklebost

**Affiliations:** 1 Department of Tumor Biology, Institute of Cancer Research, the Norwegian Radium Hospital, Oslo University Hospital, Oslo, Norway; 2 Department of Molecular Cell Biology, Institute of Cancer Research, the Norwegian Radium Hospital, Oslo University Hospital, Oslo, Norway; 3 Faculty of Medicine, Centre for Cancer Cell Reprogramming, Institute of Clinical Medicine, University of Oslo, Oslo, Norway; 4 Centre for Molecular Medicine Norway (NCMM), Nordic EMBL Partnership, University of Oslo, Oslo, Norway; 5 Faculty of Mathematics and Natural Sciences, Department of Biosciences, University of Oslo, Oslo, Norway; 6 Department of Core Facilities, Genomics Core Facility, Institute of Cancer Research, the Norwegian Radium Hospital, Oslo University Hospital, Oslo, Norway; 7 Department of Clinical Science, University of Bergen, Bergen, Norway; Universite de Nantes, FRANCE

## Abstract

Sarcomas are a heterogeneous group of mesenchymal orphan cancers and new treatment alternatives beyond traditional chemotherapeutic regimes are much needed. So far, tumor mutation analysis has not led to significant treatment advances, and we have attempted to bypass this limitation by performing direct drug testing of a library of 353 anti-cancer compounds that are either FDA-approved, in clinical trial, or in advanced stages of preclinical development on a panel of 13 liposarcoma cell lines. We identified and validated six drugs, targeting different mechanisms and with good efficiency across the cell lines: MLN2238 –a proteasome inhibitor, GSK2126458 –a PI3K/mTOR inhibitor, JNJ-26481585 –a histone deacetylase inhibitor, triptolide–a multi-target drug, YM155 –a survivin inhibitor, and APO866 (FK866)–a nicotinamide phosphoribosyl transferase inhibitor. GR50s for those drugs were mostly in the nanomolar range, and in many cases below 10 nM. These drugs had long-lasting effect upon drug withdrawal, limited toxicity to normal cells and good efficacy also against tumor explants. Finally, we identified potential genomic biomarkers of their efficacy. Being approved or in clinical trials, these drugs are promising candidates for liposarcoma treatment.

## 1. Introduction

Sarcomas make up about 1% of all cancers and are divided into bone and soft tissue sarcomas (STSs). The latter group, accounting for 87% of all sarcomas, comprises more than 50 histological subtypes, and this diversity represents a significant diagnostic and therapeutic challenge. According to statistics from the National Cancer Institute (NCI) about 50% of patients with STS die within 5 years of diagnosis. Prognosis depends on the tumor subtype, but also tumor location–those in the abdomen are more difficult to completely eradicate. Due to their rarity, diversity and the limited biological mechanistic understanding, there is only moderate commercial interest to develop sarcoma-specific therapies.

Liposarcomas are the largest subtype of STS, accounting for approximately 20% of the cases. They resemble adipose tissue, with four principal histological subtypes comprising well-differentiated/dedifferentiated (WD/DDLPS), myxoid/round celled, and pleomorphic liposarcomas. Though liposarcomas may have characteristic karyotypic aberrations which help in diagnosis, those cannot yet be therapeutically exploited.

Current first-line treatment for liposarcoma is surgery, alone or in combination with radiotherapy, while chemotherapy is the option for patients with high risk of recurrence or with widespread disease. Chemotherapy for soft tissue sarcoma generally uses a combination of several conventional chemotherapeutic agents, often ifosfamide, dacarbazine and doxorubicin. Doxorubicin monotherapy is the main first-line treatment, while second-line standards are not set. Two novel drugs have been approved by FDA for the treatment of liposarcomas, although with moderate efficacy, the microtubule inhibitor eribulin [1] and the DNA alkylating drug trabectedin [2].

Personalized medicine focuses on the correlation of drug responses with molecular features which could be used as biomarkers for patient stratification. In case of liposarcomas, several studies on their genome landscapes have been done, but mainly two characteristic molecular markers are known, amplification of the 12q13-15 region (including *MDM2*, *FRS2* and usually also *CDK4*), which is detected in almost all WDLPS or DDLPS, and the fusion oncogenes FUS-DDIT3 or EWSR1–DDIT3 in myxoid and round cell liposarcoma. Those markers are used mainly for diagnosis, although trials on targeting MDM2 [3] and CDK4 [4] have been performed with limited success, and further clinical trials with such inhibitors are under way. A systematic effort to find molecular drivers of STS was done by Barretina and colleagues, who tried to identify novel subtype-specific genomic alterations in, among others, 95 liposarcoma patient samples [5]. They identified mutations in PIK3CA in myxoid/round cell liposarcoma and TP53 and NF1 in pleomorphic liposarcoma. Although those mutations might help to identify tumors that might be responsive to PI3K or mTOR inhibitors, they were only present in some patients. Moreover, a clinical trial with a dual PI3K/mTOR inhibitor has shown no association of responses with PIK3CA mutations [6]. More recently, The Cancer Genome Atlas soft tissue sarcoma study confirmed low mutation burden in DDLPS and identified JUN amplification as a potential marker of poor survival in a subset of samples. They also showed recurrent deletions of ATRX and CDKN2A in a minority of samples but pointed out the importance of overall methylation status as a biomarker in DDLPS [7].

Drug sensitivity screens provide a different, phenotype-based approach to identification of new anti-cancer therapies (reviewed in [8]). The NCI-60 [9], Cancer Cell Line Encyclopedia [10] and Genomics of Drug Sensitivity in Cancer [11] projects have made collections of data from approximately 1000 cell lines. Those huge, collaborative efforts report not only sensitivities of those cell lines to multiple drugs, but also correlate drug sensitivity to genomic features. Unfortunately, none of those databases comprise liposarcomas. Even a sarcoma-specific drug screen of 63 sarcoma cell lines contained just two liposarcoma cell lines [12]. Just recently, the first multidrug screening of 3 myxoid liposarcoma cell lines was published, showing the survivin inhibitor YM155 to limit tumor growth [13].

In this work we have used medium-scale high-throughput drug sensitivity screening on a panel of liposarcoma cell lines to identify compounds that broadly target liposarcoma. This approach identified several candidate drugs that are presently not used for sarcoma treatment, but which *in vitro* were generally more efficient than the currently used anti-sarcoma chemotherapeutics in the tested concentration ranges.

## 2. Materials & methods

### 2.1 Cell lines and culture conditions

SW872 and SA-4 (both classified as “liposarcoma”) were purchased from ATCC. Pleomorphic liposarcoma cell lines: LiSa-2 was provided by P. Möller [14] and LS2 by D. Broccoli [15]. Cell lines from dedifferentiated liposarcomas: LPS141, LPS510 and LPS853 (all DDLPS) were received from J. Fletcher [16], GOT3 from Åman [17] and NRH-LS1 derived in-house [18]. Cell lines from well-differentiated liposarcomas: FU-DDLS-1, was provided by J. Nishio [19] and T449, T778 and T1000 were gifts from F. Pedeutour [20]. Cells were cultured in RPMI-1640 medium (#R0883) supplemented with 10% FBS, 1% L-Alanyl-L-Glutamine (all Sigma-Aldrich, St. Louis, USA) and grown at 37°C, 5% CO_2_. Immortalized human mesenchymal stroma cells iMSC#3 [21] were cultured in minimum essential medium alpha medium (#32561029, ThermoFisher Scientific) with 20% FBS and L-Alanyl-L-Glutamine. Short tandem repeat (STR) DNA profiling was performed on all cell lines and their identity confirmed. Cells were confirmed negative for mycoplasma using the VenorGeM Mycoplasma Detection Kit (Minerva Biolabs, Berlin, Germany).

### 2.2 Drugs

The anti-cancer compound library (#L3000) was purchased from Selleck Chemicals (Munich, Germany) pre-dissolved in DMSO (Sigma-Aldrich, Missouri, USA). Actinomycin D (#A9415) and melphalan (#M2011) were from Sigma Aldrich (St. Louis, USA) and trofosfamide (#T892000) from Toronto Research Chemicals (Canada). Individual drugs for validation and ED50 experiments were purchased from Selleck Chemicals and dissolved in DMSO or ethanol according to the manufacturer’s recommendation. For each drug treatment the appropriate control was used with a DMSO/ethanol concentration corresponding to that of the highest drug concentration.

### 2.3 Drug screen and data analysis

349 compounds from anti-cancer Selleck Chemical library were pre-aliquoted at five concentrations, increasing in 10-fold steps from 1 nM to 10 μM, in 384-well plates using an Echo Liquid Handler. Each plate also contained positive (benzethonium chloride, BzCl) and negative (DMSO only) controls. Additional drugs were added manually to the same plates at the same concentration range. Cells were seeded directly into the plates containing the drugs. After 72h viability was assessed through levels of ATP, with Cell Titer Glow assay (Promega, Wisconsin, United States) and luminescence quantified using a fluorometer. Data from drug screens were analyzed with R version 3.4.0 (R_Core_Team, 2017) normalized to median positive (BzCl) and median negative (DMSO) controls using formula: (drug-BzCl)/(DMSO–BzCl), with total viability calculated as the area under the curve over all five drug concentrations, adjusted using qualitatively constrained quantile smoothing splines, R package ‘cobs’ version 1.3–3 [22]. The raw values of positive (BzCl) and median negative (DMSO) controls on each assay plate were aggregated and used to calculate z’-factors, as a measure of assay performance and data quality, with a z’-factor > 0.5 representing acceptable data. For two cases where the z’-factor was below 0.5, strictly standardized mean difference (SSMD) was additionally calculated, giving the population value (β) of SSMD -4 and -5, respectively, indicating good quality [23]. Data visualization was done in R using ‘ComplexHeatmaps’ package version 1.20.0 [24]. The same assay was also used when assessing viability of non-tumor cells and tumor explants.

### 2.4 Cell proliferation assay

The cellular proliferation rate was measured using the IncuCyte ZOOM live-cell imaging system (Essen Bioscience, Birmingham, UK) with the corresponding software application (version 2013BRev1). First the growth rate for each cell line was established to estimate the seeding numbers of cells corresponding to 15–30% of confluence at the start of the experiment and maximum 70% after 72h. The drug treatment was initiated approximately 18h after seeding. The proliferation rate was measured as cell confluence over time through the acquisition of photographs under phase contrast every third hour for the entire duration of the drug treatment. During the drug treatment, media were not changed, and cells were not split. The growth reducing drug concentration was defined as lowest drug concentration leading to significant decrease of cellular growth between 40 and 72h after start of treatment. To determine that concentration, plots were generated using IncuCyte software and differences in growth between 40 and 72h were calculated using least-square means with the ‘emmeans’ R package version 1.4.3 [25]. Growth arresting concentrations were defined as minimal drug concentration under which cell confluence between 96 and 120h increases by less than 3%. Though growth arrest occurred much earlier, these time points ensured that the growth arrest is a stable phenomenon. These growth arresting concentrations were used for drug withdrawal, apoptosis and cell cycle experiments. All the data were verified with the plots. The presented values correspond to median values from two (T449, all compounds) to five (all cell lines, triptolide) biological replicates.

### 2.5 Viability assay for concentration-response curves and GR50 calculations

Cell viability was measured using the CellTiter-Glo Luminescent Cell Viability Assay (Promega, Wisconsin, United States). For each experiment the same cell numbers were seeded as for the proliferation experiments. Seven drug concentrations were used, starting from 100μM with 10-fold dilutions and also a vehicle control appropriate for each drug (DMSO or ethanol). After 72h of drug or vehicle control treatment levels of ATP were measured with CellTiter-Glo. The data from six to nine biological replicates, each with three technical replicates were collected. A nonlinear regression model with different four-parameter log-logistic function was used for modelling of the drug dose-response using ‘GRmetrics’ R package version 1.8.1 [26]. The calculated GR50 metric is equivalent to the IC50 metric but takes into account the growth rate of cells. Therefore, it estimates better the effect of the compound on cell viability and allows for comparison between different cell lines with different growth rates.

### 2.6 Apoptosis assay and cell cycle assay

Cells were treated with drugs at the concentrations corresponding to previously established growth arresting concentrations and collected between 17 and 72h. Time of collection was individual for every drug/cell line combination; cells were collected after the beginning of complete growth inhibition (based on the growth curves data) but only when first signs of morphological change or cell death were visible (estimated microscopically approximately every 2h). After methanol fixation, cells were barcoded with different combinations of Pacific Blue/Pacific Orange dyes (Thermo Fisher Scientific) for different drug treatments. After mixing, cells were incubated in the TUNEL reaction mix for 1h at 37°C, according to the manufacturer’s instructions (Roche, Germany), stained with Streptavidin-Cy5 conjugated Ab and digested with RNAse. Cells were counter-stained with propidium iodide and analyzed using LSR II Flow Cytometer (BD Biosciences, NJ, USA. The data were processed with FlowJo Version 7.6.5 (Tree Star, Ashland, USA). The gating strategy is presented in Fig 3A. Cy-5 positive cells (as compared to control sample) were calculated as apoptotic. Cell cycles of non-apoptotic cells were analyzed using the built in FlowJo Cell Cycle Analysis module. Three independent biological replicates were done for each cell line/drug combination with exception of FU-DDLS-1 and T1000 –for which we only had two replicates available.

### 2.7 Explant cultures

Explants of tumors from patients with liposarcoma were made using Tumor Dissociation Kit and gentleMACS Dissociator (both Miltenyi Biotec, Germany) according to the manufacturer’s instructions. Cells were used either directly after isolation or at passage 1—after seeding into cell culture flasks for a brief period to remove contaminating blood cells. Patients’ consents and project were approved by the Regional Committee for Medical Ethics in Southeastern Norway (Project 17866). To isolate primary MSC, bone marrow aspirate was obtained from a healthy donor, mononuclear cells were isolated using a Ficoll density gradient (Lymphoprep TM, Stemcell Technologies Inc.). Cells were suspended in Mononuclear Cell Medium (MCM; PromoCell) supplemented with 100 units/mL of penicillin and 100 μg/mL of streptomycin, adherent bone marrow-derived blast cells grown as monolayer culture. Mononuclear leukocytes were isolated from blood using the same procedure without selection of adherent cells.

### 2.8 RNA-Seq data generation and pre-processing

RNA was isolated using the AllPrep DNA/RNA Mini Kit (QIAGEN, Venlo, Netherlands). mRNA sequencing libraries were prepared using 100 ng of total RNA and the Illumina TruSeq Stranded mRNA Library Prep kit following supplier’s instructions. The libraries were sequenced on a NextSeq 500 Illumina sequencer using a High Output v2 kit chemistry, generating 2x75 bp paired-end sequence reads. RNA-Seq reads were aligned using STAR aligner (v.2.5.0b) against the human reference genome (UCSC hg19, RefSeq & Gencode gene annotations), and FPKM estimation was generated by Cufflinks 2 using the RNA-seq alignment app at Illumina BaseSpace. Two genes were represented twice. For these duplicates, we selected the values of the gene with the highest standard deviation. Finally, the FPKM values were quantile normalized using the *qnorm* function in Bioconductor package *preprocessCore* [27]. A small number (1e-5) was then added to the data to allow for log transformation, after which the data were then log_10_ transformed. This resulted in a matrix containing expression values for 26,226 genes and 13 liposarcoma cell lines. The RNA-seq data have been submitted to NCBI with the accession number PRJNA662692.

### 2.9 Correlation of drug responses with expression of single genes in the drug-connected process

We chose genes from the pathways/processes known to be connected with the identified drugs. For the gene list, see S1 Table. After removing data with FPKM values below 10 for all the cell lines, GR50 drug response scores were correlated with chosen gene expression values using Spearman correlation. Genes with (|Spearman ρ|)≥0.65 and, at the same time p-value below 0.05, were considered correlated.

### 2.10 Identification of potential drug response biomarkers

To obtain a set of genes that were reproducibly associated with drug response, and that may be potential biomarkers of drug response, we performed a one-leave-out cross validation on the Spearman correlation analysis involving all genes. For each of the 6 drugs, we performed a total of 13 analyses, each time leaving out one sample (one specific cell line) from the analysis. We then selected those genes that were consistently associated (|Spearman ρ|)>0.8 across all of the 13 cross-validations) with drug response.

### 2.11 Correlation of drug response with gene expression signatures

We correlated GR50 drug response scores with gene expression values using Spearman correlation. We next performed pre-ranked Gene Set Enrichment Analysis (GSEA) [28] on the Spearman correlation coefficients, using Reactome pathways [29] from MSigDb version 6.2 [30] filename “c2.cp.reactome.v6.2.symbols.gmt”. We defined pathways with significant over-representation of high correlation values using stringent FDR (FDR < 0.001) and GSEA Enrichment Score (|ES| > 0.5) thresholds.

## 3. Results

### 3.1. Identification of candidate sarcoma treatments by drug screening

**Fig 1:**
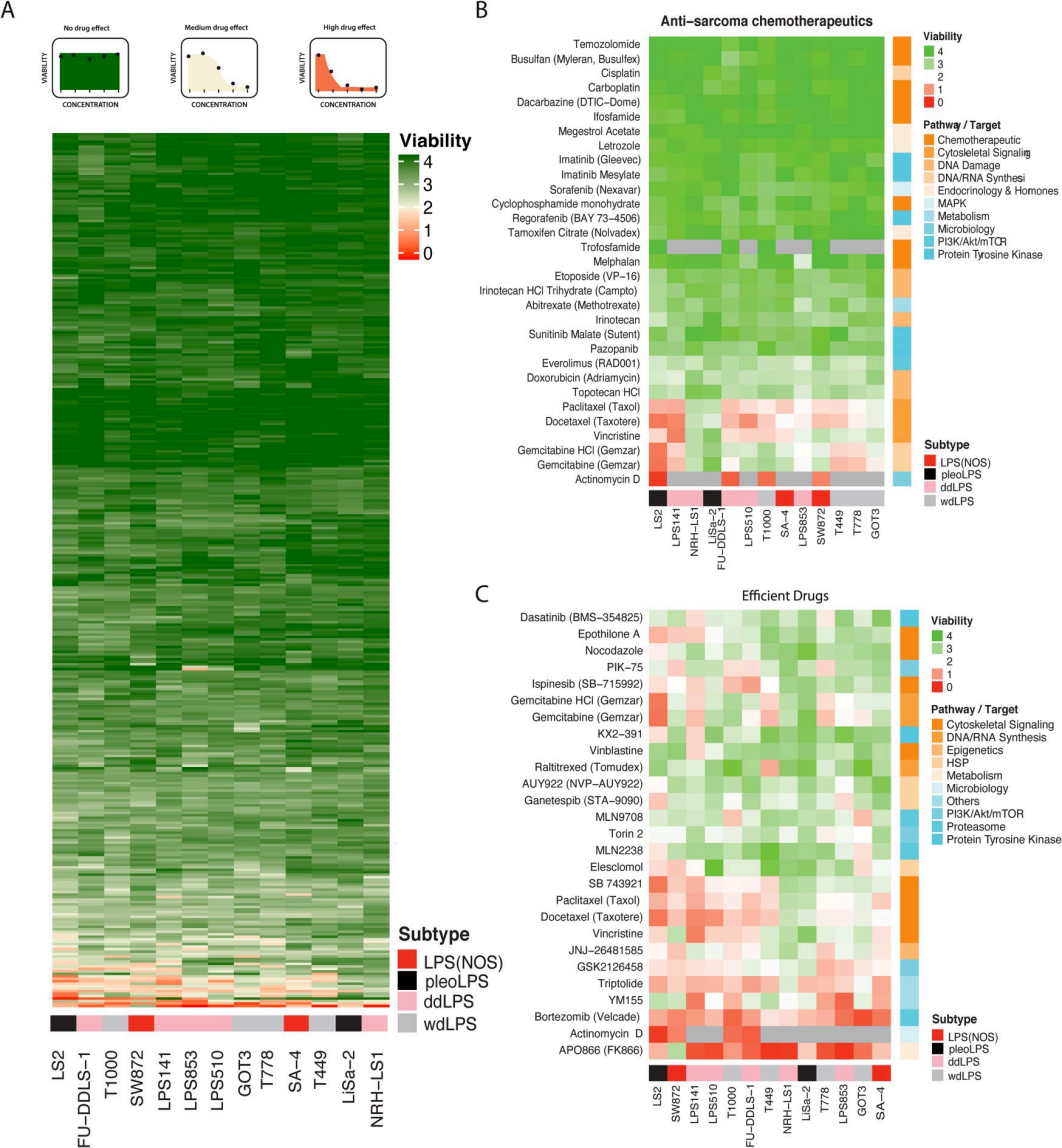
Visualization of drug screen results. The heatmap colors reflect area under the response curve from viability data, for the combined used concentrations, green—highest, red–lowest, as illustrated by examples above the heatmap. Grey color–not tested. **A,** Unsupervised clustering of liposarcoma cell lines (columns) and tested compounds (rows), according to sensitivity patterns. The compounds cluster into several, mainly inactive (green), groups. **B,** Heat map of cell lines clustered by response to chemotherapeutic drugs in clinical use for sarcoma, showing no clear association by subtype of origin or with expected pathways/targets of the drug (corresponding color bar on the right). **C,** Heatmap of cell lines clustered by response to the 25 most efficient compounds from the drug screen (reducing combined viability to at least 50% for at least one cell line). Also here there is no clear association with subtype of origin. The LPS subtypes are indicated above their names, LPS(NOS)–not otherwise specified: red, pleoLPS: black, ddLPS: pink, and wdLPS: grey.

Thirteen liposarcoma cell lines representing dedifferentiated, well-differentiated, pleomorphic and unspecified subtype liposarcomas were grown in 384-well format in the presence one of the 353 compounds, each at five 10-fold increasing concentrations (from 1 nM to 10 μM) for 72h, after which their viability was scored by measuring ATP levels. The viability was normalized to positive and negative controls and was calculated as sum of viabilities obtained with all the five used concentrations. That means if, e.g., the compound was killing cells at all the tested concentrations, the viability value would be 0, and if the compound was not killing cells at any tested concentrations, the value would be 4. If the compound was reducing the viability to 50% at 100 nM, and to 0% at 1 and 10 μM, the total viability would be calculated as 2 (see scheme on the top of Fig 1A). Within the tested concentration range, the majority of the drugs had very little effect on viability regardless of the target or pathway (Figs 1A and S1). The drug screen included 28 chemotherapeutic drugs currently in clinical use in Norway for the treatment of sarcoma. Of those, only gemcitabine, a DNA synthesis disruptor, actinomycin D, an inhibitor of transcription, and cytoskeletal disruptors vincristine, paclitaxel and docetaxel had significant impact on viability in the concentration range used in our assay, but without ant clear subtype-specific profiles (Fig 1B). However, it is important to note that clinically relevant concentrations for those compounds, *e*.*g*. maximum concentration achieved in plasma (C_max_), are often higher than the upper range of our screen– 10 μM.

As a proof-of-concept for potential STS therapy, we chose to investigate in more detail novel drugs likely to act on different mechanisms and broadly across the cell panel. The most efficient drugs were chosen based on the decrease of cell viability to at least 50% for the combined used concentrations for at least one of the cell lines, but also here without apparent subtype-specific pattern (Fig 1C). We decided to further investigate 6 drugs: MLN2238 –a proteasome inhibitor, GSK2126458 –a PI3K/mTOR inhibitor, JNJ-26481585 –a histone deacetylase inhibitor, triptolide–a multi-target drug, YM155 –a survivin inhibitor, and APO866 (or FK866)–a nicotinamide phosphoribosyl transferase (NAMPT) inhibitor. The selected compounds have been previously investigated in some sarcoma cell lines, but not in liposarcoma (Table 1). All are in clinical trials, but not against sarcomas. Notably, MLN2238 has recently been registered for a liposarcoma clinical trial but is not recruiting patients yet. Bortezomib and ispinesib were two other top candidates from the screen (Fig 1C), which we decided not to analyze in further detail as they are already in clinical trial for sarcoma (see clinicaltrials.gov and [31]).

**Table 1:** Characteristics of the selected compounds.

Compound	Target	Clinical trials*	Used for sarcoma?**
APO866	blocking NAD^+^ production from salvage pathway, inhibitor of nicotinamide phosphoribosyl transferase (NAMPT)	3 (Phase 1/2) CTCL, BCLL, melanoma	Ewing sarcoma cell lines [32]
Triptolide	a diterpene derived from Tripterygium wilfordii Hook f., a Chinese medicinal herb, reported to inhibit cell proliferation and induce apoptosis; multiple mechanisms proposed	10 (Phase 2) Crohn’s disease, HIV, polycystic kidney disease Minnelide advanced gastrointestinal carcinoma, pancreatic cancer, AML, advanced solid tumors	fibrosarcoma and osteosarcoma cell lines and xenografts [33–36]
GSK2126458	dual inhibitor of PI3K and mTOR	3 (Phase 1) Advanced Solid Tumors, colorectal	osteosarcoma cell lines rhabdomyosarcoma [37]
JNJ-26481585	inhibitor of HDAC, anticancer activity critically depends on an intact mitochondrial pathway of apoptosis	6 (Phase 2) advanced solid malignancies and lymphoma, lung, ovarian, myeloma, leukemia	rhabdomyosarcoma and synovial sarcoma cell lines [38,39]
YM155	inhibitor of anti-apoptotic protein survivin (BIRC5)	11 (Phase 1/2) melanoma, breast, NHL, prostate, lung, advanced non-small cell lung carcinoma and other solid tumors	rhabdomyosarcoma, osteosarcoma, Ewing sarcoma, synovial sarcoma, myxoid liposarcoma and MFH/UPS cell lines [13,40–43]
MLN2238	inhibits the 20S proteasome	89 (Phase 3)	osteosarcoma cell lines [44]

*clinicaltrials.gov, status of 02.01.2020

**only first reports shown.

### 3.2. Verification of response to the selected drugs

Having identified several candidate drugs, we decided to analyze their effect on cell viability with higher resolution by measuring cell viability over 7 drug concentrations, allowing for calculation of the concentrations at half maximum effect corrected for cellular growth rate (GR50, [45] S2 Fig). Cell viability was determined after 72h incubation for the complete panel of the liposarcoma cell lines. Although the GR50s (Table 2) differ between the cell lines and drugs, overall, the values were in sub-micromolar range. The results largely confirm those from the primary screen (Fig 1C), with APO866 and triptolide being the most effective while MLN9708 and GSK2126458 least effective for the majority of the cell lines. Surprisingly, JNJ-26481585 was more efficient here than in the screen as compared to GSK2126458, which might reflect drug quality batch effects, or different ways of calculating the response for the drug screen and the GR50s. Selectivity towards specific cell lines was also confirmed for APO866, MLN9708 and to a lesser extent, YM155. For these three drugs, the screens correlated with GR50 results with Spearman ρ of 0.75, 0.79, and 0.53, respectively. There was no linear correlation between GR50 values, and the response values calculated from the drug sensitivity screen in case of the other three drugs. For triptolide, the drug with the lowest correlation, this was likely because response values were very close in range, both for the screen (average difference between best and worse responding cell line 1.2 as compared to the median of 1.7 for all the drugs) and for the GR50 calculations (difference between best and worse responding cell line 11nM as compared to the median of 275 nM). In case of GSK2126458 and JNJ-26481585, the main responses remained comparable. For GSK2126458, NRH-LS1 was one of the worst responders, both in the screen and in the GR50 calculations, while LPS853 and T778 remained relatively sensitive to the drug. Similarly, for JNJ-26481585, LPS141 and NRH-LS1 having the highest GR50 values were also most resistant in the primary screen, while T778 was sensitive in both experiments. Some discrepancies were also observed, such as the sensitivity of T449 and T1000 towards GSK2126458, or sensitivity of T449 towards JNJ-26481585, which were not observed in the primary drug screen. This might represent either a false negative result of the drug screen, or it could be due to importance of growth rate in the mode of action of those drugs. Taken together, these data confirm the results from the high-throughput drug screen (Table 2 and Fig 1C).

**Table 2:** Half maximum effective concentration corrected for cell growth (GR50, in nM) for all the drugs (rows) and liposarcoma cell lines (columns).

Compound and its clinically relevant concentration*	LS2	T1000	FU-DDL S-1	GOT3	LPS 853	LPS 141	SW 872	LPS 510	SA-4	LiSa-2	T449	NRH-LS1	T778
APO866 150–400	8	6	1	5	1	6	4	3	10	8	1	1	3
JNJ-26481585 1500–140	94	10	12	22	18	102	70	33	44	21	25	103	25
MLN9708 30–300	629	463	635	99	95	522	553	841	325	745	1031	743	774
GSK 2126458 100	110	14	96	28	50	182	401	82	86	54	10	469	52
Triptolide 14	6	8	8	7	5	12	2	5	13	9	8	7	8
YM155 17	358	17	49	187	4	11	2	93	30	234	712	69	96

*values show Cmax or Css (italics).

### 3.3. Growth inhibitory effects of selected compounds on liposarcoma cell lines

For the experiments presented above, the viability of the cell cultures was determined using CellTiter-Glo, which is a combined measure of cell number and metabolic state. To more precisely measure cell numbers, we directly imaged cells by time-lapse microscopy. Representative growth curves for each cell line and each compound at all concentrations tested are shown in S3 Fig.

**Fig 2:**
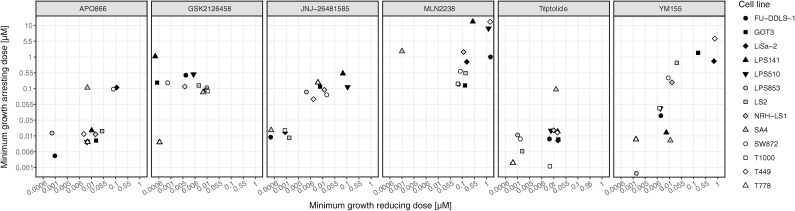
Growth inhibition profiles of the selected compounds. Minimum growth reducing drug concentration (between 40 and 72h, in linear phase) was plotted against the concentration completely inhibiting the cells growth (between 72 and 120h). Since the plotted values are derived from a discreet set of data (a series of 10-fold drug concentrations), the *jitter geom* function from ggplot2 [46] was used in R for better visualization. The function spreads the data in such a way, that the plotted points do not overlap each other, but are slightly set apart, meaning they do not exactly correspond to the concentrations on the axis, but their original values belong to the closest cross-point of the shown concentrations. Values represent median of 3 biological replicates.

By plotting the drug concentration that led to complete growth inhibition between 96h and 120h of drug treatment against the lowest drug concentration that reduced cell growth between 40 and 72h, the diversity of the responses of the tested cell lines to the six compounds can be visualized (Fig 2). The coordinates along the y-axis indicate the efficiency of the compound, and if the x-coordinates are similar, the compound works in a narrow concentration range, immediately exerting full growth inhibitory effect. APO866 and triptolide appear to work in this manner, where the first drug concentration affecting cell growth also results in complete growth arrest. Other compounds first slow down cell proliferation at lower concentrations before completely arresting it at higher doses, so the drug response is more stepwise with cell proliferation progressively decreased with increasing compound concentration.

Fig 2 shows that triptolide and APO866 were the most efficient compounds, arresting the majority of the cell lines already at 10 nM, consistent with the observed GR50s. Similarly, the least effective compound MLN9708 required at least 1 μM to completely arrest more than half of the cell lines tested. YM155 and MLN9708 had the widest range of growth inhibitory concentrations from 1 nM to 10 μM in the different cell lines. Such varied responses indicate that predictive biomarkers should be explored to better identify possible responders. GSK2126458, and to a lesser extent APO866 and triptolide, permanently arrested cell growth at the same concentration for at least half of the cell lines, suggesting they act broadly against liposarcoma cells. The growth inhibitory concentrations correlated well with both GR50s and the drug screen data for APO866, MLN9708 and YM155. That indicates that growth arrest in fact represents a reduction in cell viability. For the other three compounds, GR50 values and concentrations arresting growth do not correlate well (S4 Fig). However, JNJ-26481585 clearly divided cells into two groups, largely overlapping with the trend seen in the GR50 for the best and worst responders. In case of triptolide and GSK2126458, the lack of correlation was likely due to the quite similar and therefore not easily distinguishable response values of growth inhibitory concentrations. Overall, combined assessment of growth arresting concentrations and GR50s shows an extended picture of responses to the tested compounds, indicating, among others, their specificity and mode of action.

### 3.4. The mechanism of drug-induced growth arrest

**Fig 3:**
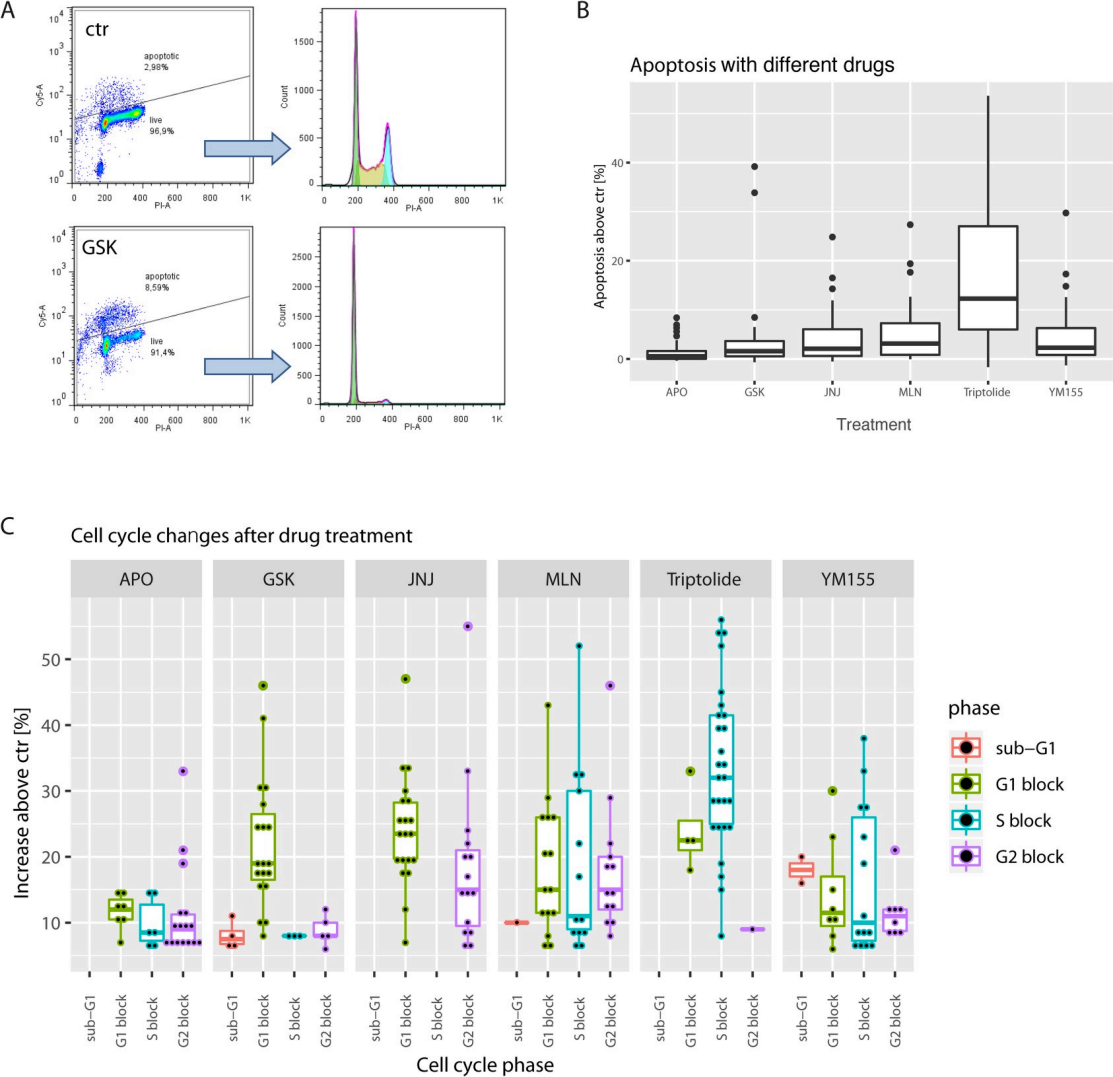
Mechanism of action of the selected compounds. A, gating strategy for the selection of apoptotic cells. After selection of singlets, gates for apoptotic fraction were established based on the vehicle treated cells (ctr) and applied to the drug treated cells (here GSK) from the same experiment. Top–apoptotic cells, bottom–live cells used for cell cycle determination. B, boxplots show increase in % of apoptotic cells in drug treated cells’ populations. Boxplot’s center line visualizes the median, the box the IQR, and the lines 1.5xIQR. Data for all the cell lines were pooled together. C, cell cycle distribution after drug treatment. Data for all the cell lines were pooled together. Boxplots show increase in cell cycle phases as compared to the ctr cells shown, dots represent individual experimental points.

Since cell growth inhibition can be the consequence of multiple effects we determined, if the observed growth arrest was mainly a result of apoptosis or cell cycle inhibition. Cells were treated with the compounds at growth arresting concentrations and collected when the first signs of apoptosis and/or cell morphology change were microscopically visible. This strategy allowed us to observe a snapshot of the mechanism of the investigated drugs at one specific time window. Apoptosis was determined with TUNEL assay and percentage of apoptotic cells was counted and compared to the control. The non-apoptotic fractions were used to determine cell cycle changes. Fig 3A demonstrates the gating strategy. The results showed that triptolide induces strong apoptosis (above 15%), JNJ-26481585, MLN9708 and YM155 induce moderate apoptosis (above 5%) while APO866 and GSK2126458 did not induce apoptosis above 5% for majority of cell lines (Fig 3B). Fig 3C shows results for the cell cycle analysis. For clarity, only the data points with more than 5% increase in any of the cell cycle phases are included. Triptolide is mainly blocking the cells in S phase, GSK2126458 in G1 phase and JNJ-26481585 in G1 and G2 phases. APO866, MLN9708 and YM155 lead to less specific arrest. Overall, in the initial phase, APO866 does not induce apoptosis but non-specific cell cycle arrest, consistent with previous reports showing that NAMP inhibitors induce oncosis and not apoptosis [47]. JNJ-26481585 induces apoptosis and arrests the cells outside the S phase. GSK2126458, while not inducing apoptosis, blocks the cells in G1 phase. MLN9708 and YM155 induce both apoptosis and non-specific cell cycle arrest, while triptolide is a strong inducer of S phase arrest and apoptosis for all cell lines tested.

### 3.5. The reversibility of drug-induced growth arrest

**Fig 4:**
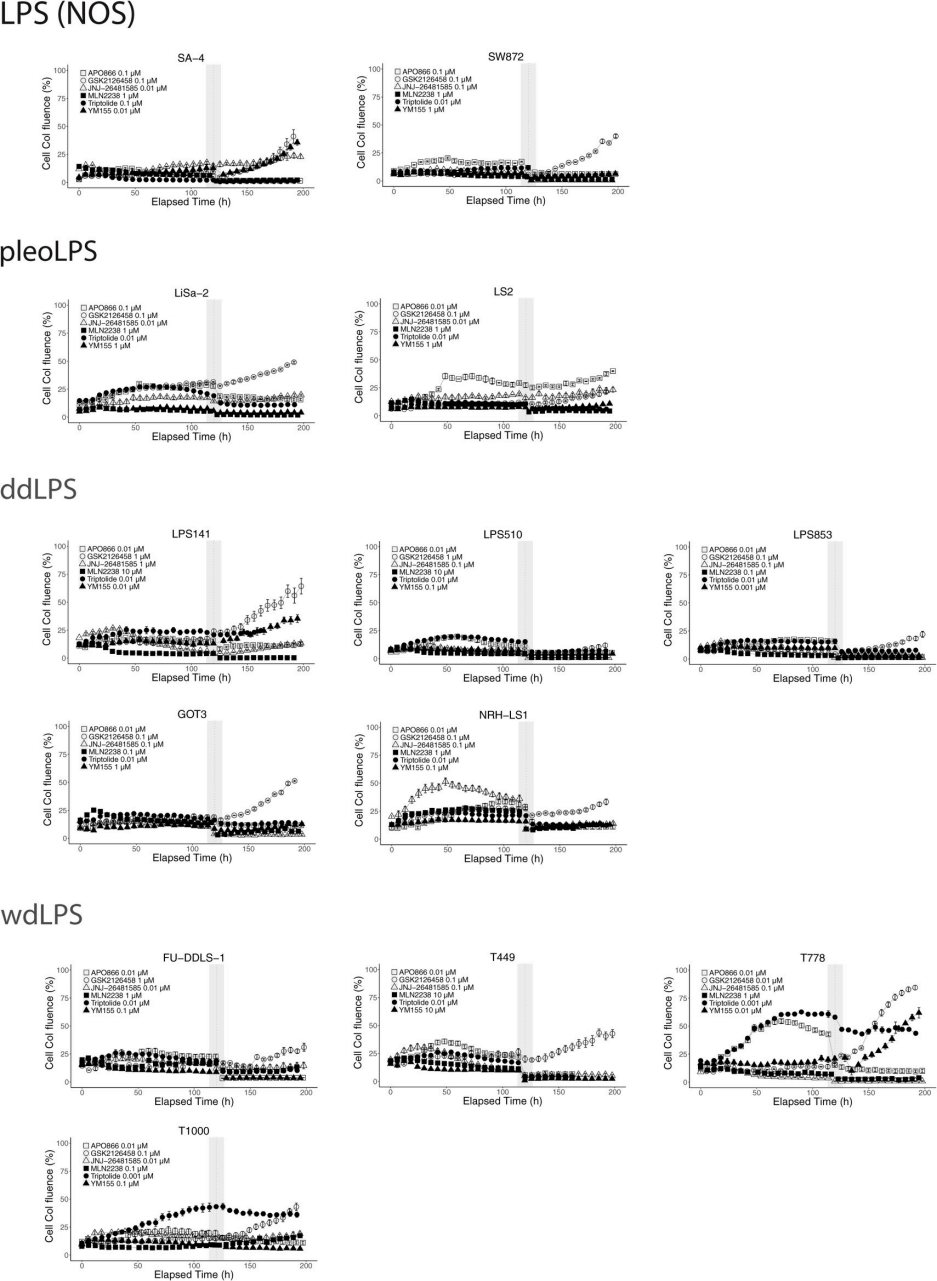
Reversibility of growth inhibition. Cells were treated with the indicated concentrations of the compounds for 120h, after which the compound was removed (vertical grey bar) and cells were grown for additional 72h. Drug concentrations tested correspond to the lowest concentration shown to completely inhibit cells growth between 72 and 120h. In some cases, the cells regained the growth capability after drug withdrawal, especially for GSK2126458. Shown representative of 3 biological replicates, error bars represent the standard error of the mean (SEM) of technical replicates.

An important prerequisite for clinical implementation of these compounds as treatment for liposarcoma is irreversibility of the induced growth inhibition. We thus assessed if the drugs were only transiently cytostatic, which is especially important in case of the compounds that seem to work mainly via cell cycle arrest (APO866, GSK2126458). Cells were treated with the compounds at growth arresting concentrations for 120h after which the compounds were removed and cell numbers were monitored for another 72h. APO866, triptolide, JNJ-26481585 and MLN9708 irreversibly inhibited growth of all tested cell lines within that period (except for cell line T778 with MLN9708) (Fig 4). Some cell lines such as LPS141, T778 and SA-4 resumed growth after removal of YM155. However, the growth of those cell lines could be irreversibly inhibited, if a 10x higher concentration of YM155 was used (S5 Fig). All of the tested cell lines regained their growth rate within 3h after the withdrawal of GSK2126458, explaining the apparent lack of concordance of GSK2126458 GR50s with growth inhibition data. To irreversibly arrest cell proliferation GSK2126458 had to be used at 10x higher concentrations (100x for GOT3) (S5 Fig). Those concentrations are above physiological ranges (see Table 2), and these effects should be accounted for assessing use of GSK2126458 in clinic. These data indicate that the majority of the compounds can induce irreversible cell growth arrest.

### 3.6. The selected compounds are moderately toxic to normal cell cultures

**Fig 5:**
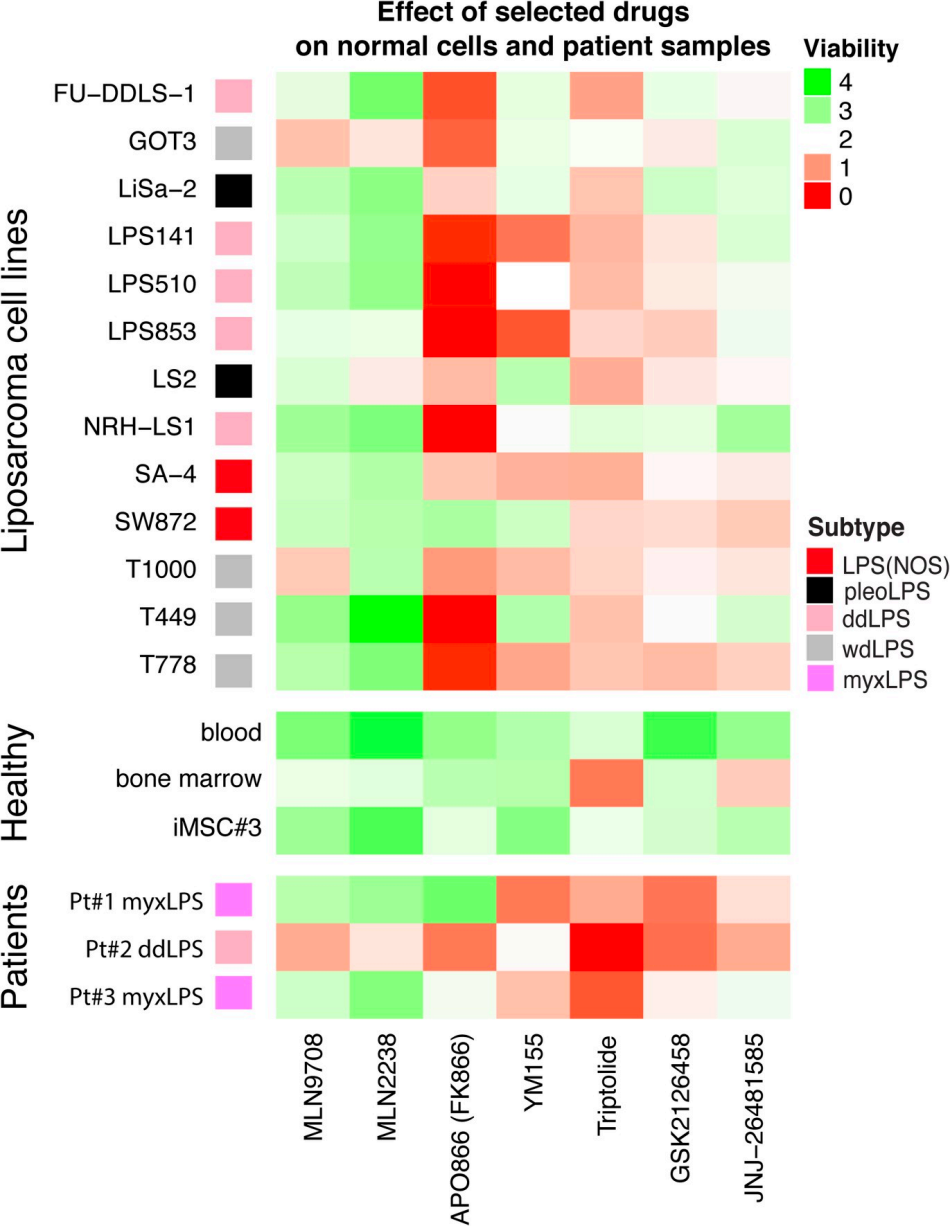
Toxicity of the compounds towards cells isolated from fresh tumors of liposarcoma patients and normal cell cultures. Viability of liposarcoma cell lines, normal cells represented by blood, cells from healthy donor bone marrow, and immortalized non-tumorigenic mesenchymal stroma cell line iMSC#3, and patient samples (from patient 1, 2 and 3) is given, using the same drug concentrations and analysis as for the drug screen. Compounds are less toxic for normal cells than for liposarcoma cell lines with notable exceptions of triptolide and, in some cases JNJ-26481585, which were more toxic for bone marrow cells. Heatmap colors represent normalized cell viability calculated for the combined used concentrations (area under curve (AUC)) for each drug, green—highest, red—lowest.

To evaluate the therapeutic windows, we investigated the toxicity of the compounds towards normal cells *ex vivo*, such as blood and bone marrow from healthy donors, as well as on a non-tumorigenic immortalized mesenchymal stroma cell line iMSC#3 [21]. After isolation from donors, blood and bone marrow cells were incubated *in vitro* for 72 h with drugs at the same concentration range as in the previous drug-screening experiments. Subsequently, cell viability was scored by measuring ATP levels. Despite some differences between cell cultures, all compounds were generally less toxic to normal cells. Two notable exceptions were triptolide and JNJ-26481585, which were more toxic to bone marrow cells *in vitro* than to liposarcoma cell lines (Fig 5, top and middle panels). Since bone marrow MSCs provide the hematologic stem cell niche [48], potential clinical implementation of these drugs as sarcoma treatment should include close monitoring of the patient’s hematological status.

### 3.7. Efficacy against ex vivo cultures of patient-derived tumor cells

Next, we tested the selected candidate drugs on cancer cells isolated from primary liposarcoma tumors that were available during the study (Fig 5, bottom panel). Two of these were from myxLPS, which was not among the cell types investigated, except perhaps one of the LPS(NOS) lines. Cells were isolated from patient material and seeded directly onto plates with pre-aliquoted drugs to the same concentration range as in the previous drug-screening experiments. Cell viability was assessed by measuring ATP after 72h. Importantly, each of the proposed drugs efficiently targeted also these primary tumor cells, although we did observe some variation between drugs and patients. Myxoid liposarcoma (patient 1 and 3) responses were more similar to each other and the LPS(NOS) cell line SW872 than to the dedifferentiated liposarcoma (patient 2), which on the other hand is more similar to the ddLPS cell line GOT3 and wdLPS line T1000. Triptolide was most efficient for all the samples, while responses to APO866 were most varied. Of note, the ddLPS cells from patient 2 were most sensitive to all of the tested compounds. We observed good response to YM155, concordant with a previous study on myxLPS [13], but also the general sensitivity pattern was promising also for this subtype. Taken together, while the group of tested samples from patients was small, these data support the activity of the compounds against tumors isolated from patients.

### 3.8. Biomarkers of sensitivity towards selected compounds

In the next step, we looked for predictive gene expression biomarkers for responses to the selected drugs. We correlated GR50 values with mRNA expression of known targets of the researched compounds and genes in their associated pathways. Since high GR50 means low sensitivity, negative correlation (ρ < 0) indicates that high gene expression was associated with response to the compound.

Looking first at the expected immediate targets of the drugs, there was little correlation between expression and response. For YM155 we did not see correlation between response and expression of survivin, nor the response to MLN2238 and expression of proteasome components or expression of histone deacetylases with sensitivity to JNJ-26481585. The exception was the expression of *HDAC1*, which correlated with sensitivity to the latter compound (ρ = -0.70). We were also unable to correlate the expression of most of the genes from the PI3K and mTOR pathways with the response to GSK2126458 with the exception of tumor suppressor *TSC2* (ρ = -0.74). GSK2126458 is expected to work in cells with altered PI3K signaling, and mutations in *PIK3CA* or *NF1* have been shown to be some of the few mutations identified in soft tissue sarcomas [5]. However, lack of correlation between PI3K status and results from a clinical trial indicate a much more complicated relation [6]. For APO866, we found inverse correlation between expression of *NAMPT* and sensitivity (ρ = 0.67), confirming previous findings for other NAMPT inhibitors [47]. This correlation, however, needs to be taken with caution, since GR50 values for APO866 in all the cell lines were within the same order of magnitude. Nevertheless, in general, it is conceivable that high target expression requires more drug for complete inhibition, so these observations do not challenge the current mechanistic models, that only require the target to be present and active.

Because we identified very few predictive biomarkers for drug response among the known targets of the compounds, we performed a genome-wide search for drug response biomarkers, investigating correlations of drug response with the expression of all 26,226 genes using Spearman correlation across the 13 cell lines. To identify reproducible expression patterns, we performed leave-one-out cross-validation analyses by repeating the correlation analysis of gene expression with drug response 13 times, each time leaving out a different cell line. We performed this analysis for each drug to obtain a set of robust, reproducible biomarkers (S6A Fig). We identified 2–16 genes for each of the six drugs that associated with resistance, or sensitivity. The identified top biomarkers included, not surprisingly, high *STAT2* expression for response to GSK2126458 consistent with JAK/STAT signaling leading to activation of the PI3K/mTOR pathway [49]. Other top biomarkers predicted response to histone deacetylate inhibitor JNJ-26481585, including GYS1, involved in glycogen metabolism, known to be reprogrammed in cancer cells. The mechanism likely involves fueling glycolysis, known to play an important role in histone acetylation [50]. For triptolide, expression of the lncRNA *MIR100HG*, encoding regulators of cell proliferation [51], positively correlated with response to the drug. In case of proteasome inhibitor MLN2238, we noted correlation of response to the drug and *STK26* expression, which is a known activator of cancer progression autophagy [52]. Overall, for each compound, we have found several candidate genes positively or negatively correlating with response to the compound which could be further investigated as response biomarkers.

Finally, we looked more globally at the level of pathway activity in the RNA-seq data for the 13 liposarcoma cell lines. We performed Gene Set Enrichment Analysis (GSEA) based on the Spearman correlations between the GR50 and the expression levels of all genes using Reactome pathways to detect, if any pathways were significantly enriched (FDR<0.001, absolute GSEA Enrichment Score >0.5) for genes correlating with the treatment across the 13 cell lines. We visualized these results in a heatmap (S6B Fig). In total, we found significant associations of 45 different Reactome pathways with at least one drug (out of a total of 659 Reactome pathways). Pathways involved in DNA replication, mitosis, and cell cycle were enriched for biomarkers of poor response to JNJ-26481585 and triptolide, suggesting that highly proliferative cells were more resistant to these drugs. Similarly, immune-related pathways, including interferon signaling and antigen processing, were enriched for biomarkers of poor response to MLN2238 and YM155. Expression of pathways with functions in protein translation predicted good response to these two drugs. Interestingly, pathway correlations of sensitivity to MLN2238 and YM155 were very similar, indicating that these drugs are likely to target similar cellular states. Activity of genes associated with respiratory electron transport and mRNA splicing predicted poor response to APO866. Finally, the sensitivity to GSK2126458 was correlated with expression of pathways involved in respiratory electron transport and DNA strand elongation and polymerase switching, indicating that high activity of such pathways may result in a better response to treatment.

## 4. Discussion

Although *in vitro* experiments using tumour-derived cell lines can reveal clinically relevant drug sensitivities, one should be aware of the inherent limitations. The artificial growth conditions and lack of host interactions and also the selection of atypical cells that can grow fast under these conditions can affect the results and threaten the translational value for clinical use. Traditionally, promising *in vitro* data would first need validation *in vivo* on patient-derived tumour xenografts in mice (PDXes), and then in phase II trials on relevant patient cohorts. This long and expensive testing phase delays and prevents new drugs for orphan diseases like sarcoma, both due to their limited "market value", and because it is demanding to accrue the number of patients needed for traditional trials. However, a number of exciting initiatives have been started, in which rational drug targets using already approved drugs can be tested in small, precisely defined, cohorts even based on laboratory data.

Our focus here on anti-cancer compounds already approved or investigated in patients can thus contribute to improved therapies, since toxicities, and thus patient risk, are generally well known.

Our cell line panel has limitations though, with some lines being stated as "not otherwise specified" liposarcoma, with no documentation (SW872 and SA-4), only two representing pleomorphic LPS (LiSa-2 and LS2), and no myxoid LPS lines. The largest subset of 9 WD/DDLPS lines is better documented, but since our repeated effort to make PDX and cell lines from WDLPS have failed, we suspect that all these rather reflect cells with DDLPS properties selected from samples diagnosed as WDLPS.

Based on sensitivities found in our drug screen, we selected 6 compounds which were not previously used for the treatment of liposarcoma: the proteasome inhibitor MLN2238, the PI3K/mTOR inhibitor GSK2126458, the histone deacetylase inhibitor JNJ-26481585, the multi-target drug triptolide, the survivin inhibitor YM155, and the NAMPT inhibitor APO866. Importantly, YM155 was during this work shown to have activity against both our explants of myxLPS cells from patient samples (Fig 5), confirming the finding reported previously [13]. GSK2126458, YM155 and APO866 have been used in the Genomics of Drug Sensitivity in Cancer Project where genomic features correlating with drug sensitivity have been determined in cells from some other sarcoma types [11]. Interestingly, in that study, the presence of the Ewing sarcoma-specific EWSR1-FLI1 fusion protein correlated with sensitivity to each of those compounds, which might be mechanistically relevant to sensitivity also in other sarcomas although lacking this specific fusion gene. No other statistically significant correlations to other cancer types or genomic features were found for these drugs in that study. Triptolide has been tested within the NCI-60 project and showed an average IC50 of 12 nM for all cancer cell lines, which is within the range observed in our experiments. The proteasome inhibitor MLN2238 and histone deacetylase inhibitor JNJ-26481585 were not investigated in the above-mentioned broad drug screens.

Our data show that the most efficient and promising compounds among the selected six were triptolide and APO866. Triptolide is an active ingredient of the Chinese medicinal herb, *Tripterigium wilfordii*, which has been used to treat rheumatological and immunological diseases. Since the isolation of the active compound in 1972, many different activities have been demonstrated, including anti-neoplastic potential (reviewed in [53,54]). Its anticancer properties have been demonstrated in animal xenograft models and prodrug formulations overcoming its poor aqueous solubility has entered clinical trials [55]. However, a phase I trial with the semi-synthetic derivative F60008 and multiple animal studies showed multiple organ or tissue damage and sometimes morbidity, therefore minnelide, a less toxic, water-soluble derivative of triptolide is currently in clinical trials for cancer [54,56,57]. Also here we saw some toxicity towards bone marrow cells. Interestingly, following exposure to triptolide, accumulation of p53 occurs by suppression of MDM2, reducing degradation of p53 and leading to increased induction of p21, and then apoptosis [58]. This is in line with previous studies blocking MDM2 by Nutlin in liposarcomas, and may partly explain the good responses [18,59,60]. At the same time, a more complicated mechanism is probably at play, since we did not observe any correlation of response to triptolide with MDM2 expression in our cell line panel (ρ = 0.34, p>0.2). The maximum therapeutic plasma concentration (C_max_) of triptolide in humans is around 0.15–0.4 μM, well above the GR50 values obtained in our study [61].

Another potent drug candidate for liposarcomas, APO866, is an anti-metabolic drug which induces cell death by specifically inhibiting the biosynthesis of NAD^+^. NAD^+^ is essential for cellular metabolism, protein modification and mRNA synthesis, and as such not subject to commonly known mechanisms of multidrug resistance. A clinical trial of 24 patients with advanced solid malignancies refractory to standard therapies, including two sarcomas, reported mean steady state serum concentration (C_ss_) of 14 nM (comparable to concentrations used here), with tolerable adverse effects and a suggestion of a clinical benefit (no objective responses, although four patients had stable disease) [62]. However, other trials have shown dose-limiting toxicities with NAMPT inhibitors and no objective responses likely due to insufficient drug doses (reviewed in [47]). A mitigation strategy for the toxicity has been proposed, where normal cells could be protected by the NAPRT1-dependent NAD salvage pathway which is often inactivated in cancer cells, if nicotinic acid is administered together NAMPT inhibitor [63].

JNJ-26481585 was very efficient but also had some toxicity towards bone marrow cells in our study. However, the adverse event profile of JNJ-26481585 when tested in patients with solid tumors was comparable with that of other HDACi [64]. Moreover, in 10% of heavily pretreated patients, JNJ-26481585 treatment resulted in partial response or stable disease. With a dosing and treatment schedule recommended for phase II clinical trials, the measured C_max_ was within 1.5–14 nM, comparable to our GR50 results.

MLN2238 is a proteasome inhibitor with improved pharmacodynamic profile and antitumor activity *in vitro* and *in vivo* compared to bortezomib [65]. A phase I study of intravenous MLN2238 showed manageable safety profile, limited antitumor activity and C_max_ of up to 1400 nM at maximum tolerated dose [66]. The C_max_ from that study was higher than the GR50s from our data, and C_ss_ for the same dose was within 30–300 nM, which is comparable to our data.

YM155 is an inhibitor of survivin, a member of the inhibitor of apoptosis (IAP) protein family, and often is activated in various cancers. Phase I clinical trials with YM155 in patients with advanced solid malignancies or lymphoma showed complete or partial responses for 14% of patients. C_ss_ measured at maximum tolerated dose was 17 nM, well comparable to our GR50 values for sensitive cell lines [67]. We confirmed the activity in myxoid liposarcoma explants, supporting the testing of this drug in liposarcoma patients.

The dual inhibitor of PI3K and mTOR, GSK2126458, was used in phase I trial of multiple tumor types (sarcoma, kidney, breast, endometrial, oropharyngeal, and bladder cancer). C_max_ was around 100 nM, which is above the GR50 values for our responding cell lines. Durable objective responses were observed; however, they were not associated with *PIK3CA* mutations [6]. Though clinical trials and our data comparing toxicity of GSK2126458 towards normal cells versus tumor cells indicate the compound has selective and permanent efficacy, it is important to note that we have observed only a temporary growth arrest with GSK2126458 used in the physiological range, whereas higher, and perhaps more toxic, concentrations were needed for permanent growth arrest (Table 2, Figs 4 and S5). These data are *in vitro* only, so caution needs to be taken before considering this compound for clinical use against liposarcoma.

Overall, our data support considering the selected compounds for sarcoma treatment, since they seem to offer promising therapeutic windows *in vitro* and the drug concentrations required for inhibition of cell growth and reduction of viability are within the range of clinically acceptable doses. However, since even the drugs with the broadest activity, triptolide and APO866 showed variable responses between cell lines, further emphasized by the fact that these drugs showed varying levels of toxicity in clinical trials [55,62], patient stratification is probably required and predictive biomarkers should be investigated as part of such trials. With so few samples, our search for molecular biomarkers obviously has to be exploratory. One candidate biomarker could be the inverse correlation between expression of *NAMPT* and sensitivity to its inhibitor, APO866, as observed here. In case of the other inhibitors and their targets such direct correlation biomarkers were not found, but correlation with gene expression patterns were observed. However, these *in vitro* data need to be validated in patients.

## 5. Conclusion

We have used a high-throughput drug sensitivity screen to identify novel therapeutics against liposarcomas. There was no clear specificity for certain subtypes, as might be expected, since these are drugs developed and tested in other cancer types, and not designed for specific mechanisms in liposarcomas. The identified compounds act broadly and can conveniently be repurposed, since they all are in clinical use or in clinical trials for other diagnoses. We have verified that the compounds work *in vitro* at concentrations comparable to those used clinically and target both apoptosis and cell cycle arrest. We have also showed that *in vitro*, the drugs are more efficient towards both liposarcoma cell lines and tumor explants from tumors of patients with liposarcoma than towards cells from healthy tissues. Taken together, our data support further efforts towards repurposing the compounds for liposarcoma treatment. As the next step, *in vivo* validation of the activities of these drugs on patient-derived xenograft models could be a next step before clinical trials are proposed.

## Supporting information

S1 Fig Drug screen results divided by pathway or pathway groups. Results were divided according to the pathways and clustered within each subgroup. Columns—liposarcoma cell lines, rows–tested compounds. Heatmap colors represent normalized cell viability calculated for the combined used concentrations (area under curve (AUC)) for each drug, green—highest, red—lowest. Color bar on the left indicates the pathway the drugs are affecting.
(TIF)

S2 Fig **Dose-response curves of various sarcoma cell lines after treatmen**t: **A, JNJ-26481585**, **B, MLN2238**, **C, YM155, D, GSK2126458, E, APO866**, **F, triptolide,** at range of different concentrations—from 0.1 nM to 100 μM for a period of 72h. Four-parameter log-logistic function was used for modelling of the normalized growth rate inhibition. Dots represent individual data points from six to nine biological replicates, each with three technical replicates.
(TIF)

S3 Fig Growth rate of various sarcoma cell lines after treatment with: **A, APO866**, **B, GSK2126458**, **C, JNJ-26481585, D, MLN2238, E, triptolide**, **F, YM155** with a range of different concentrations—from 0.1 nM to 100 μM for a period of 120h. One representative experiment is shown (n = 3), error bars represent the standard error of the mean (SEM) of technical replicates.
(TIF)

S4 Fig Correlation of GR50 values with drug screening results and growth arrest concentrations. Diagonal matrix represents Spearman correlation coefficients, values corresponding to the color bar on the right. Untransformed data was used for calculations. Only statistically significant correlations (p<0.05) are shown.
(TIF)

S5 Fig Irreversibility of growth inhibition. Cells which regained the growth capability after drug removal (Fig 4) were treated with the 10x highest concentrations of the compounds (100x for GSK2126458 and GOT3**)** in the same conditions as previously. None of the cell lines regained the growth capability after drug withdrawal. One representative experiment is shown (n = 3), error bars represent the standard error of the mean (SEM) of technical replicates.
(TIF)

S6 Fig Potential biomarkers of sensitivity to the selected compounds. A, Biomarkers from the one-leave-out cross-validation analysis that reproducibly associated with drug response. GSK.: GSK2126458, MLN.: MLN2238, Trip.: Triptolide. **B**, Pathways significantly associated with drug response. The color bar in the heatmap indicates log_10_ FDR values, adjusted for the sign of association: enrichment for genes correlated with sensitivity are shown in red; values with enrichment for inverse associations with sensitivity are shown in blue. FDR values greater than 0.05 appear in white.
(TIF)

S1 Table (PDF)
