# Supplementary figures and images for: Discovery of novel candidates for anti-liposarcoma therapies by medium-scale high-throughput drug screening
Source: PLoS One. 2021 Mar 10;16(3):e0248140. doi: 10.1371/journal.pone.0248140 (PMC7946228; doi:10.1371/journal.pone.0248140)

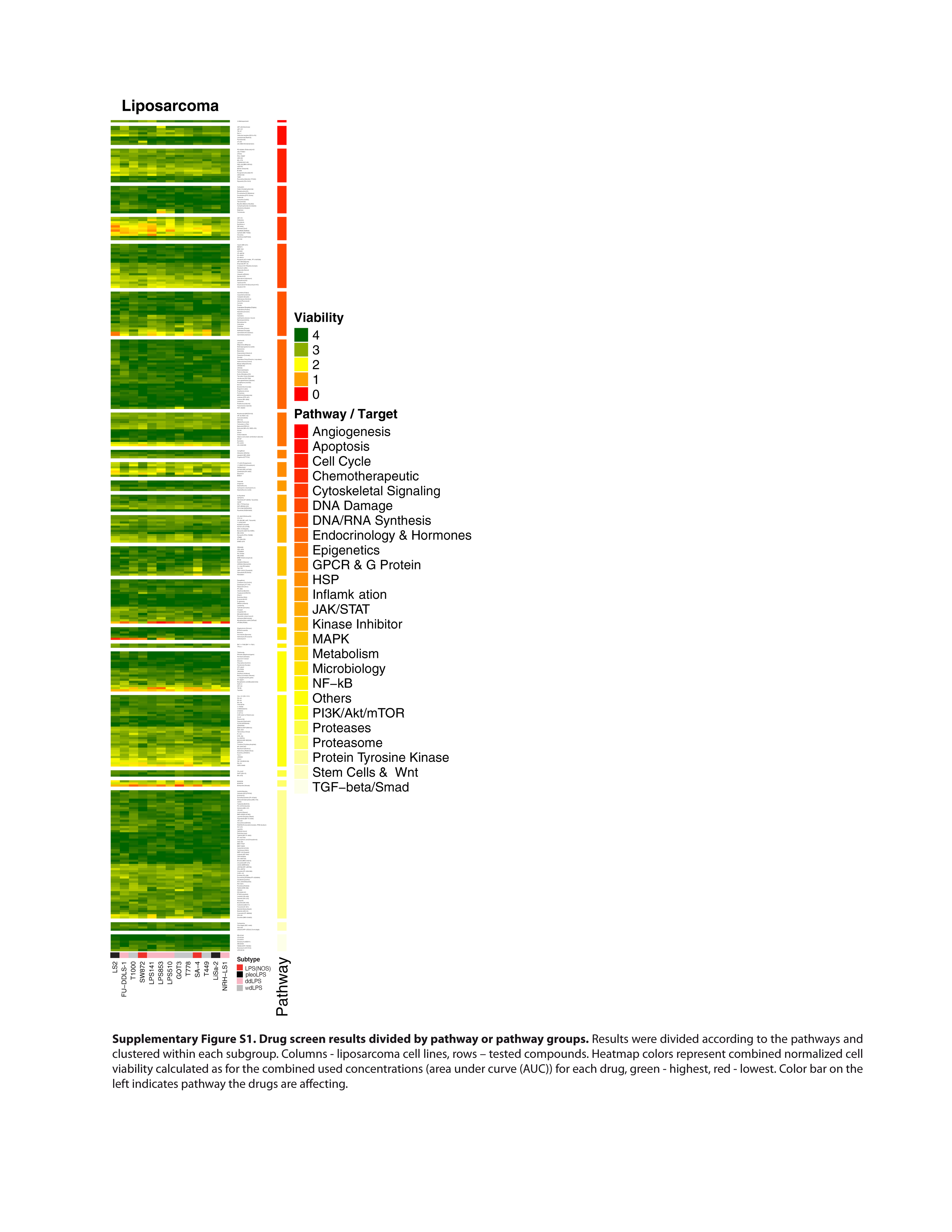

Supplement: S1 Fig — Results were divided according to the pathways and clustered within each subgroup. Columns—liposarcoma cell lines, rows–tested compounds. Heatmap colors represent normalized cell viability calculated for the combined used concentrations (area under curve (AUC)) for each drug, green—highest, red—lowest. Color bar on the left indicates the pathway the drugs are affecting. (TIF) [file pone.0248140.s001.tif]

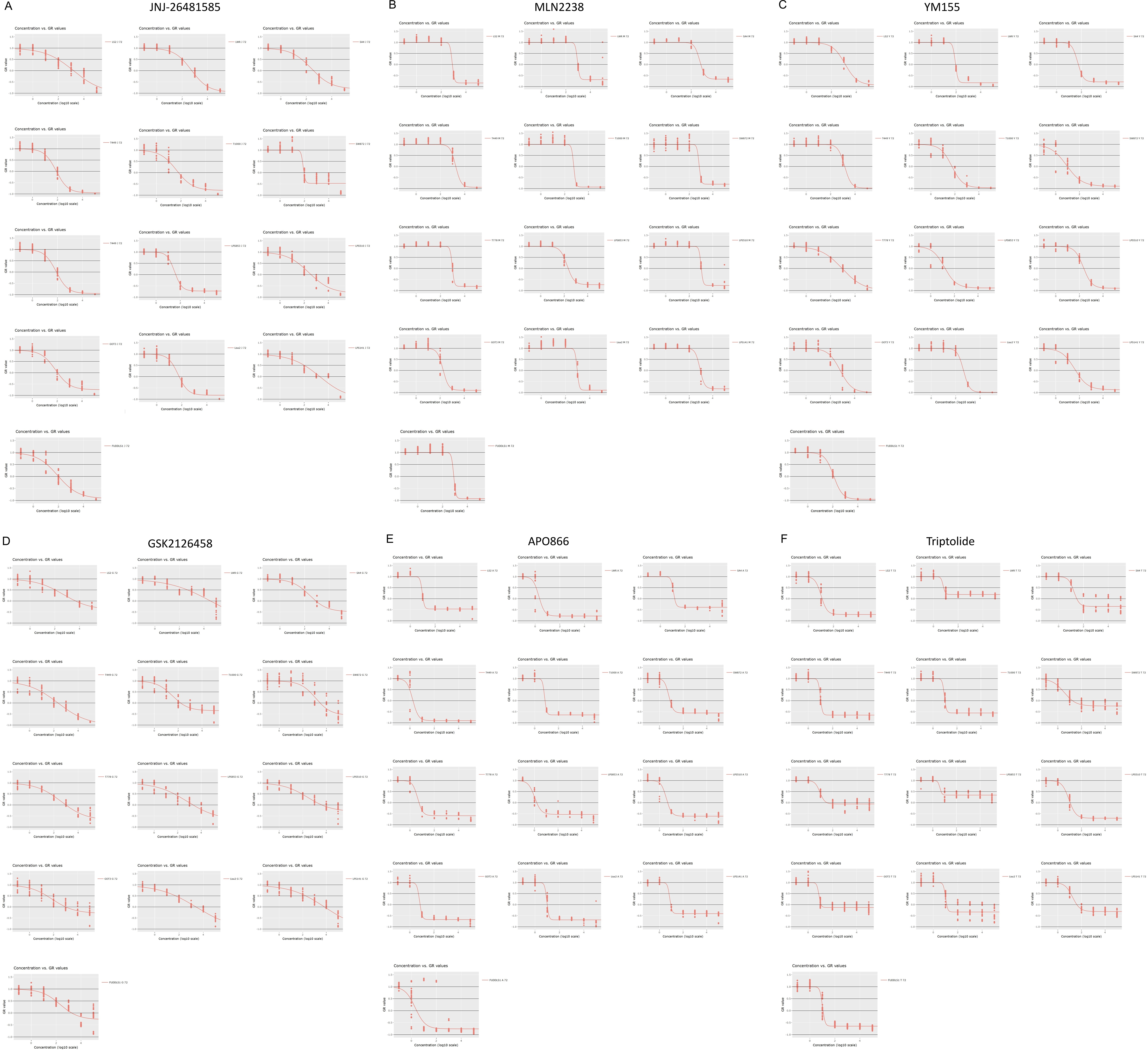

Supplement: S2 Fig — Dose-response curves of various sarcoma cell lines after treatment: A, JNJ-26481585, B, MLN2238, C, YM155, D, GSK2126458, E, APO866, F, triptolide, at range of different concentrations—from 0.1 nM to 100 μM for a period of 72h. Four-parameter log-logistic function was used for modelling of the normalized growth rate inhibition. Dots represent individual data points from six to nine biological replicates, each with three technical replicates. (TIF) [file pone.0248140.s002.tif]

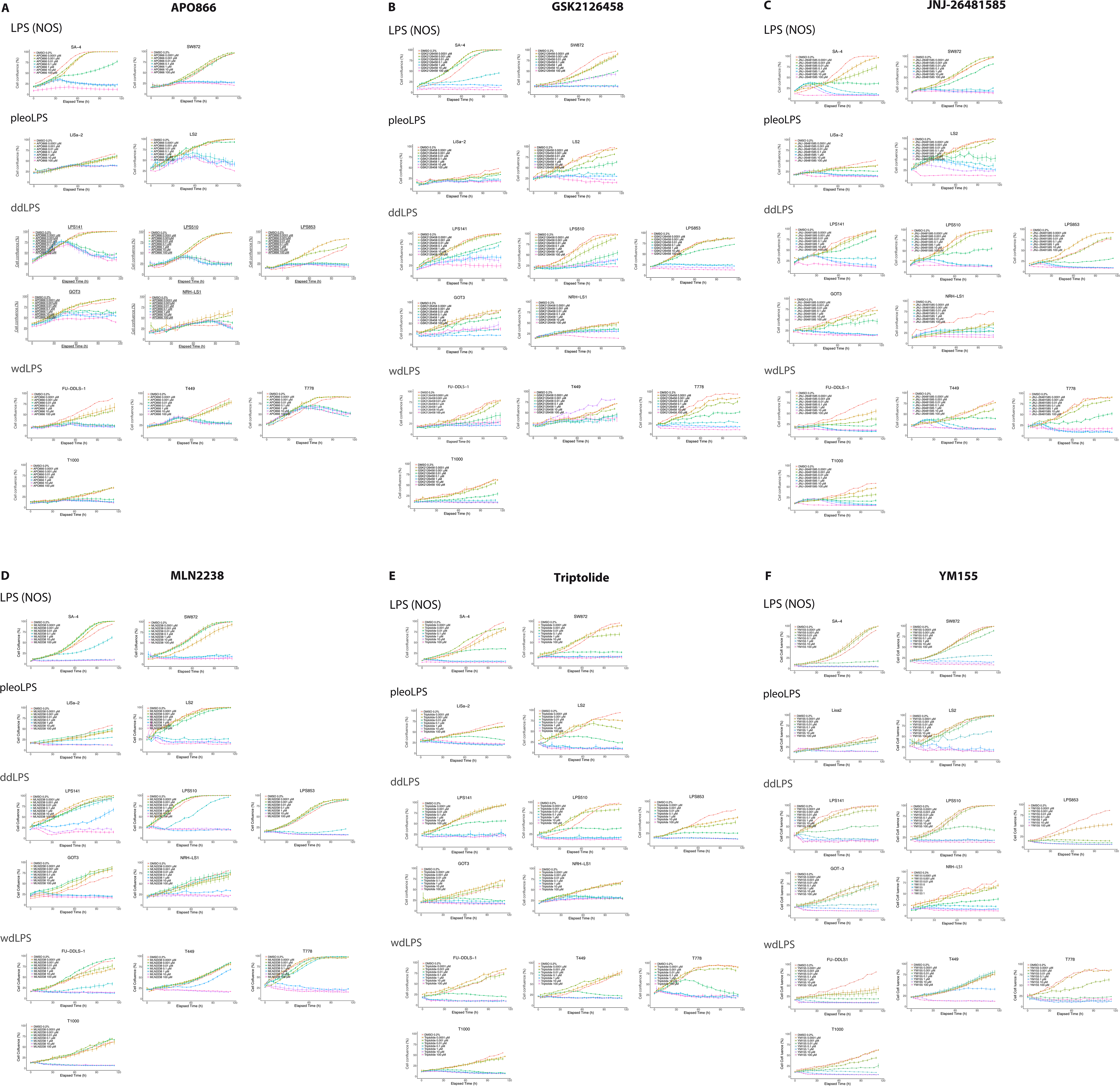

Supplement: S3 Fig — Growth rate of various sarcoma cell lines after treatment with: A, APO866, B, GSK2126458, C, JNJ-26481585, D, MLN2238, E, triptolide, F, YM155 with a range of different concentrations—from 0.1 nM to 100 μM for a period of 120h. One representative experiment is shown (n = 3), error bars represent the standard error of the mean (SEM) of technical replicates. (TIF) [file pone.0248140.s003.tif]

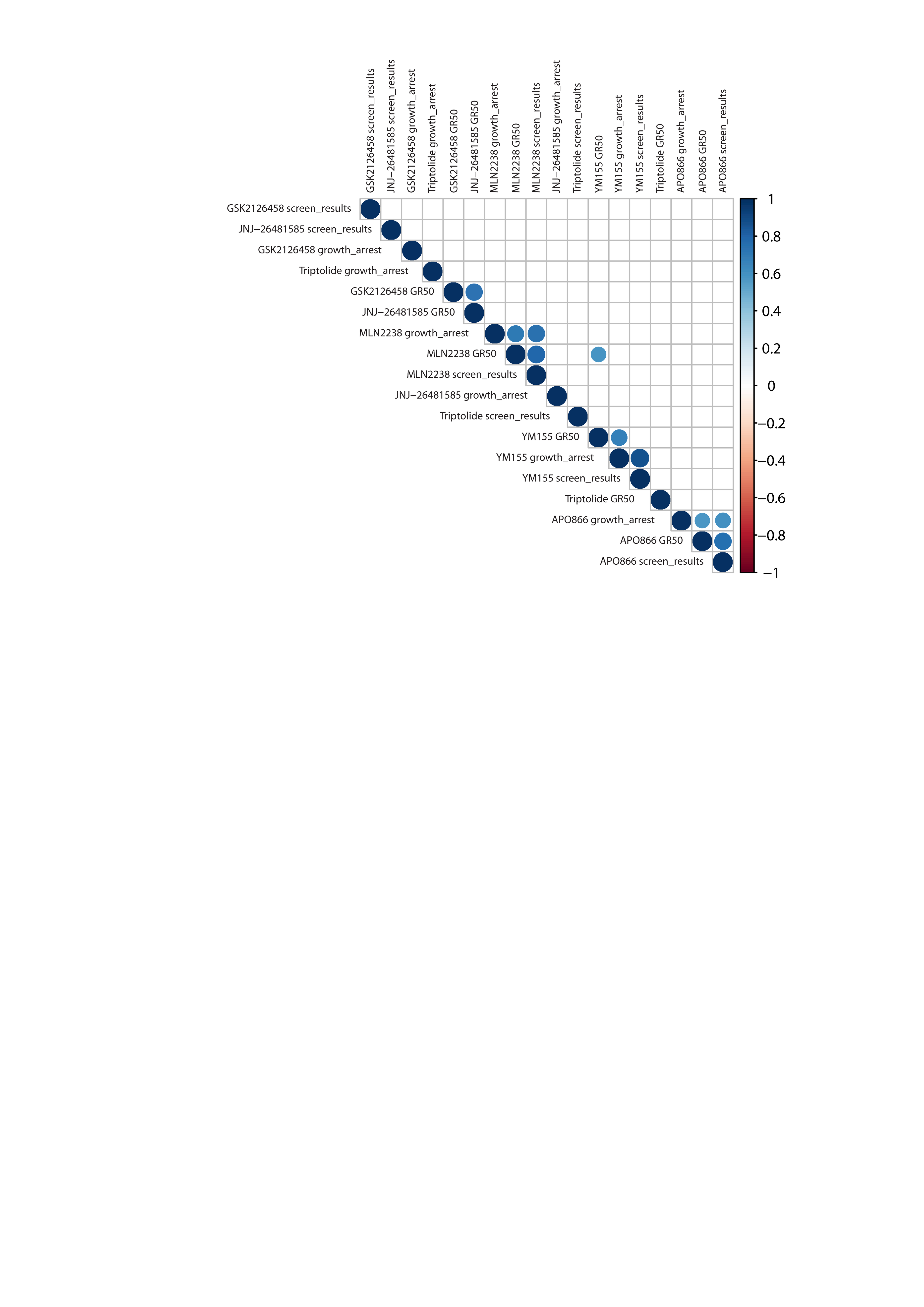

Supplement: S4 Fig — Diagonal matrix represents Spearman correlation coefficients, values corresponding to the color bar on the right. Untransformed data was used for calculations. Only statistically significant correlations (p<0.05) are shown. (TIF) [file pone.0248140.s004.tif]

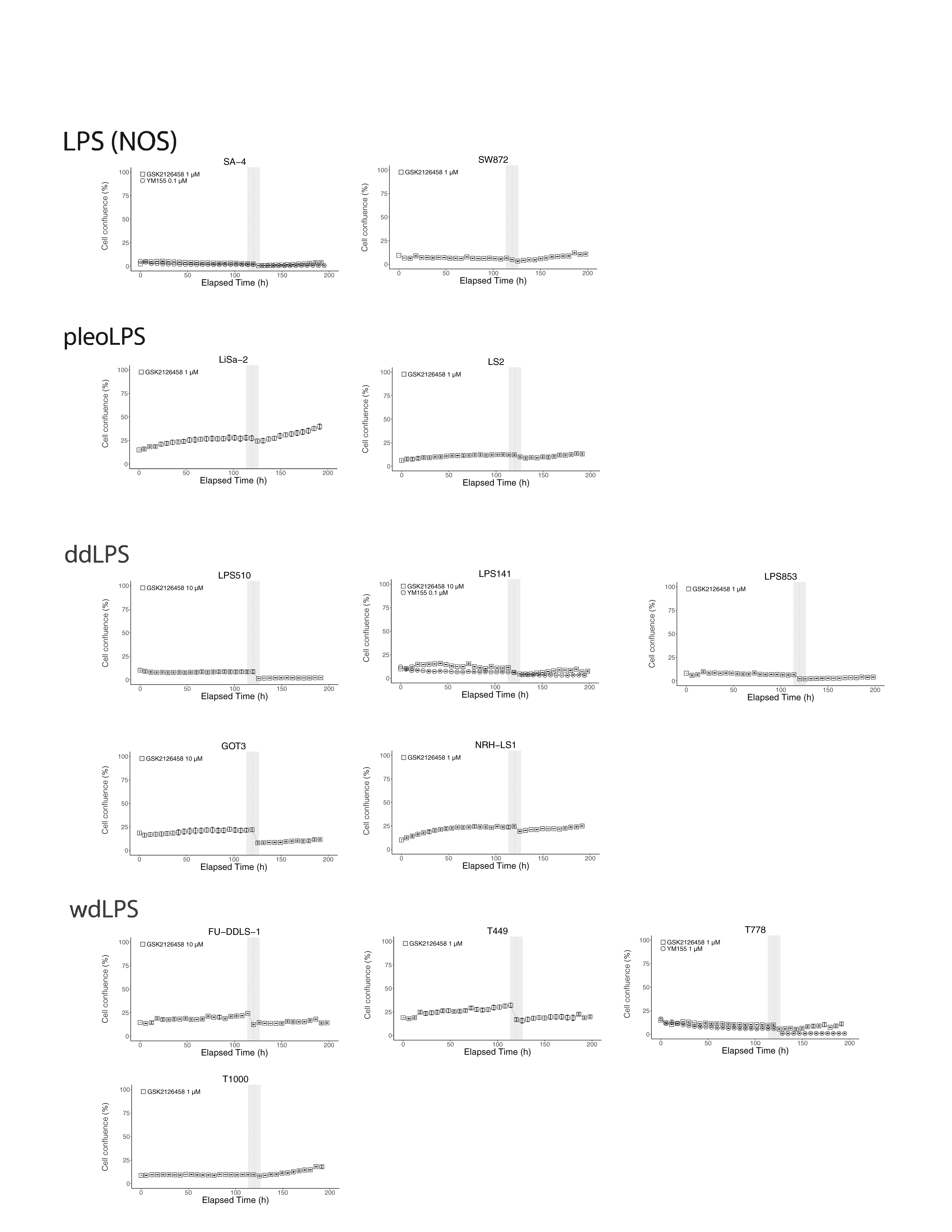

Supplement: S5 Fig — Cells which regained the growth capability after drug removal (Fig 4) were treated with the 10x highest concentrations of the compounds (100x for GSK2126458 and GOT3) in the same conditions as previously. None of the cell lines regained the growth capability after drug withdrawal. One representative experiment is shown (n = 3), error bars represent the standard error of the mean (SEM) of technical replicates. (TIF) [file pone.0248140.s005.tif]

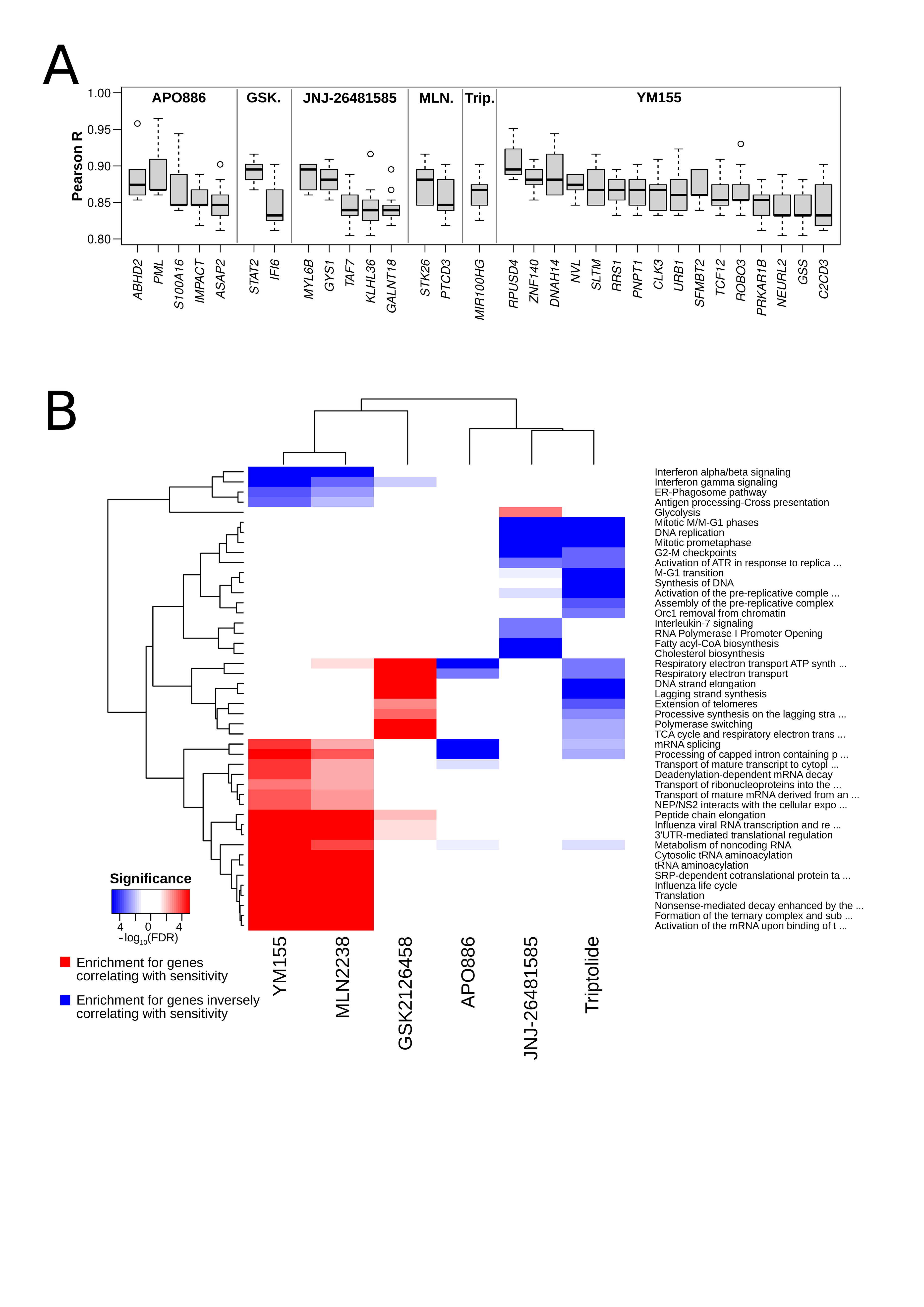

Supplement: S6 Fig — A, Biomarkers from the one-leave-out cross-validation analysis that reproducibly associated with drug response. GSK.: GSK2126458, MLN.: MLN2238, Trip.: Triptolide. B, Pathways significantly associated with drug response. The color bar in the heatmap indicates log10 FDR values, adjusted for the sign of association: enrichment for genes correlated with sensitivity are shown in red; values with enrichment for inverse associations with sensitivity are shown in blue. FDR values greater than 0.05 appear in white. (TIF) [file pone.0248140.s006.tif]
