# Supplementary material for: Discovery of novel candidates for anti-liposarcoma therapies by medium-scale high-throughput drug screening
Source: PLoS One. 2021 Mar 10;16(3):e0248140. doi: 10.1371/journal.pone.0248140 (PMC7946228; doi:10.1371/journal.pone.0248140)
Supplement: S1 Table — (PDF) [file pone.0248140.s007.pdf]

GSK21264	YM155	JNJ-26481	APO866	MLN2238	triptolide
AKT1	BIRC5	HDAC1	ACMSD	AMFR	EZH2
AKT2	SLC35F2	HDAC10	AFMID	DUSP14	SENP1
AKT3	ABCB1	HDAC11	ANKRD62P	HERPUD1	XBP1
BTK		HDAC11-AS	BST1	PSMA1	CDK7
GRB10		HDAC2	CD38	PSMA2	POLR2A
HSPB1		HDAC3	DIDO1	PSMA3	MDM2
ILK		HDAC4	IDO1	PSMA3-AS1	
MTCP1		HDAC5	IDO2	PSMA4	
PDK2		HDAC6	KYNU	PSMA5	
PIK3CA		HDAC7	NADSYN1	PSMA6	
PIK3CG		HDAC8	NAMPT	PSMA7	
PIK3R1		HDAC9	NAPRT	PSMA8	
PIK3R2			NMNAT1	PSMB1	
PAK1			PARP1	PSMB10	
PRKCA			PARP10	PSMB11	
PRKCB			PARP11	PSMB2	
PRKCZ			PARP12	PSMB3	
PTEN			PARP14	PSMB4	
TCL1A			PARP15	PSMB5	
EIF4B			PARP16	PSMB6	
EIF4E			PARP2	PSMB7	
EIF4EBP1			PARP3	PSMB8	
EIF4G1			PARP4	PSMB8-AS1	
FKBP1A			PARP6	PSMB9	
MTOR			PARP8	PSMC1	
PDK1			PARP9	PSMC2	
PDK2			PARPBP	PSMC3	
PTEN			QPRT	PSMC3IP	
RHEB			RPARP-AS1	PSMC4	
RPS6KB1			SIRT1	PSMC5	
TSC1			SIRT2	PSMC6	
TSC2			SIRT3	PSMD1	
CASP9			SIRT4	PSMD10	
CHUK			SIRT5	PSMD11	
FOXO1			SIRT6	PSMD12	
NFKBIA			SIRT7	PSMD13	
			TDO2	PSMD14	
			TIPARP	PSMD2	
			TIPARP-AS1	PSMD3	
			TNKS	PSMD4	
			TNKS1BP1	PSMD5	
			TNKS2	PSMD5-AS1	
			TNKS2-AS1	PSMD6	
				PSMD6-AS2	
				PSMD7	
				PSMD8	
				PSMD9	
				PSME1	
				PSME2	
				PSME3	
				PSME4	
				PSMF1	
				PSMG1	
				PSMG2	
				PSMG3	
				PSMG3-AS1	
				PSMG4	
				RNF139	
				RNF139-AS1	
				RNF5	
				RNF5P1	
				SEC62	
				SEL1L	
				SEL1L2	
				SEL1L3	
				UBE2G2	
				UBE2J2	
				UBXN4	
				UFD1L	
				USP14	
				VCP	
				VCPIP1	
				VCPKMT	
